# Field background odour should be taken into account when formulating a pest attractant based on plant volatiles

**DOI:** 10.1038/srep41818

**Published:** 2017-02-02

**Authors:** Xiaoming Cai, Lei Bian, Xiuxiu Xu, Zongxiu Luo, Zhaoqun Li, Zongmao Chen

**Affiliations:** 1 Key Laboratory of Tea Biology and Resource Utilization, Ministry of Agriculture, Tea Research Institute, Chinese Academy of Agricultural Science, Hangzhou 310008, China

## Abstract

Attractants for pest monitoring and controlling can be developed based on plant volatiles. Previously, we showed that tea leafhopper (*Empoasca onukii*) preferred grapevine, peach plant, and tea plant odours to clean air. In this research, we formulated three blends with similar attractiveness to leafhoppers as peach, grapevine, and tea plant volatiles; these blends were composed of (Z)-3-hexenyl acetate, (*E*)-ocimene, (*E*)-4,8-dimethyl-1,3,7-nonatriene, benzaldehyde, and ethyl benzoate. Based on these five compounds, we developed two attractants, formula-P and formula-G. The specific component relative to tea plant volatiles in formula-P was benzaldehyde, and that in formula-G was ethyl benzoate. These two compounds played a role in attracting leafhoppers. In laboratory assays, the two attractants were more attractive than tea plant volatiles to the leafhoppers, and had a similar level of attractiveness. However, the leafhoppers were not attracted to formula-P in the field. A high concentration of benzaldehyde was detected in the background odour of the tea plantations. In laboratory tests, benzaldehyde at the field concentration was attractive to leafhoppers. Our results indicate that the field background odour can interfere with a point-releasing attractant when their components overlap, and that a successful attractant must differ from the field background odour.

Plant volatiles mediate plant–insect interactions^1^,^2^. Herbivores are attracted to plant volatiles to locate food, and female herbivores also use plant volatiles to select oviposition sites^3^,^4^. Many studies have shown that only a small subset (10 compounds or fewer) of volatile compounds is used by insects for host location, although plant volatiles can contain hundreds of components^3^,^4^,^5^. Thus, a blend of few plant volatile compounds can be as attractive as host plant volatiles to herbivores^6^,^7^,^8^,^9^,^10^,^11^,^12^,^13^. Pests may recognize the host plant based on volatiles that are either species-specific compounds or species-specific blends, in which some compounds are ubiquitous and the specific proportions of compounds are important^3^,^4^. In host plant volatiles, some compounds are fundamentally important for attracting herbivores (their absence eliminates attractiveness), while others are of secondary importance (their absence reduces attractiveness) for host location^14^. A current research topic is the identification of attractive components in plant volatiles. Identifying such compounds can aid in the development of effective attractants to monitor and control insect pests in an environmentally friendly manner^15^,^16^.

Because they can be used to trap both male and female insects simultaneously, attractants based on host plant volatiles have greater potential than sex pheromones for pest management^17^,^18^,^19^. Several studies have investigated optimal combinations of host plant volatiles to improve the monitoring or trapping of insect pests^18^,^20^,^21^,^22^,^23^,^24^. Attractants based on plant volatiles have also been used successfully to manipulate the host location behaviour of target insects^25^,^26^,^27^,^28^,^29^,^30^,^31^. However, the results of laboratory tests on insect olfactory orientation often differ from those observed in field tests, and attractants that are successful in laboratory tests have not always been successful in the field^6^,^9^,^32^,^33^. Factors that can affect the success of an attractant in the field include the breadth of the target insect’s diet, the mode of insect feeding, and the complexity of the synthetic blend^31^. However, most studies have not considered the impact of the complex background odour in the field on the efficiency of the attractant.

In field environments, insects are confronted with a rich olfactory landscape that contains abundant volatiles from the main crop plant. The odour from the main crop can mask the point-releasing volatiles from attractants, thereby disrupting the attractiveness of attractants to the target pest^34^. If the components of the attractant and the crop volatiles overlap, the faint odour from 50 or 100 mg attractant (pure synthetic compounds) will not provide a distinct signal^11^. It was reported that the *Manduca sexta* moth’s ability to correctly navigate to *Datura wrightii* flower odour significantly decreased when the background odour contained benzaldehyde or creosote bush volatiles, which overlapped with the odour from *D. wrightii* flowers^35^. Likewise, mass application of a synthetic sex pheromone was shown to mask the sex pheromone released from females and disrupt the sexual chemical communication between insects^36^. Therefore, an attractant with specific components that differ from those of main crop volatiles, and the field background odour, will have a much better chance of attracting insect pests. Although there has been relatively little research on this topic, the effect of background volatile noise on attractants has been considered step-by-step in field validation tests of some attractants^18^,^23^,^32^,^37^.

The tea green leafhopper, *Empoasca onukii* (Hemiptera: Cicadellidae, previously named *Empoasca vitis*), is a cell-rupture feeder and a serious insect pest of tea, *Camellia sinensis* (L.) Kuntze (Theaceae), in China^38^,^39^. In our previous study, we found that *E. onukii* adults preferred grapevine, peach plant, and tea plant odours to clean air, and that the composition of volatiles differed among these three plant species^40^. These results raised the possibility of using peach plant and grapevine odours to develop specific attractants to monitor and manage this pest in tea plantations. In this study, our objectives were as follows: (1) to determine the attractive blends to leafhoppers in tea plant, peach plant, and grapevine volatiles; (2) to develop leafhopper-specific attractants based on grapevine and peach plant odours; and, (3) to test the efficacy and specificity of the attractants in tea plantations.

## Results

### Identification of attractive volatile blends from three plants

**Figure 1:**
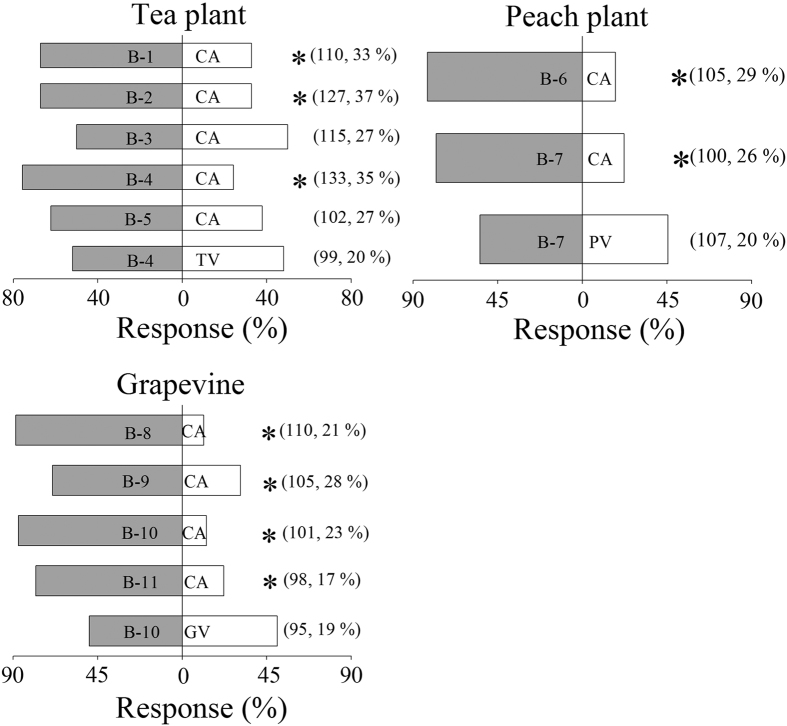
Behavioural responses of *Empoasca onukii*adults in Y-tube bioassays to 11 blends of volatile compounds (shown in Table 1). Blends were tested against tea plant volatiles (TV), peach plant volatiles (PV), grapevine volatiles (GV), and clean air (CA). Asterisks denote significant differences in attractiveness (χ^2^ goodness-of-fit test: *P* < 0.0001). Numbers in parentheses indicate numbers of tested leafhoppers followed by frequency (%) of non-responding individuals.

**Figure 2:**
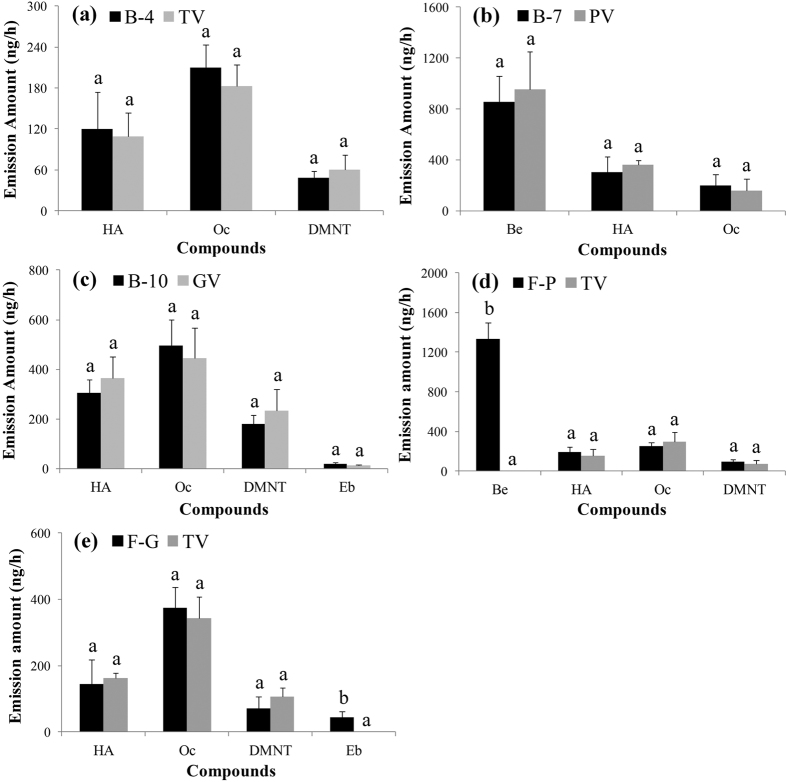
Emission amount (mean + SE, n = 4) of synthetic blends and three plant volatiles in different Y-tube tests. (**a**) Blend 4 (B-4) *vs.* tea plant volatiles (TV). (**b**) Blend 7 (B-7) *vs.* peach plant volatiles (PV). (**c**), Blend 10 (B-10) *vs.* grapevine volatiles (GV). (**d**) Formula-P (F-P) *vs.* tea plant volatiles. (**e**) Formula-G (F-G) *vs.* tea plant volatiles. B-4, B-7, B-10, F-P, and F-G were synthetic blends (see Table 1 for composition). HA, (Z)-3-hexenyl acetate; Oc, (*E*)-ocimene; DM, (E)-4,8-dimethyl-1,3,7-nonatriene; Be, benzaldehyde; EB, ethyl benzoate. Different letters on bars indicate significant differences (one-way ANOVA followed by Tukey’s multiple comparison test, *P* < 0.05).

To identify the attractive blends to leafhoppers in the tea plant, peach plant, and grapevine volatiles, the components of synthetic blends were removed one by one, and attractiveness was evaluated in Y-tube tests. The compositions of the blends are shown in Table 1. Blend 4 ((Z)-3-hexenyl acetate, (*E*)-ocimene, and (E)-4,8-dimethyl-1,3,7-nonatriene (DMNT)), blend 7 ((Z)-3 hexenyl acetate, benzaldehyde, and (*E*)-ocimene), and blend 10 ((Z)-3 hexenyl acetate, (*E*)-ocimene, DMNT, and ethyl benzoate) were significantly more attractive than clean air to leafhoppers (*P* < 0.0001) (Fig. 1). The leafhoppers could not differentiate between blend 4 and tea plant volatiles, between blend 7 and peach plant volatiles, and between blend 10 and grapevine volatiles (blend 4, *P* = 0.7503; blend 7, *P* = 0.2858; blend 10, *P* = 1) (Fig. 1). In Y-tube tests, the emission amounts of compounds in blends 4, 7, and 10 were, respectively, similar to those in tea plant, peach plant, and grapevine volatiles (*P* > 0.05) (Fig. 2a–c). Therefore, blends 4, 7, and 10 represented the attractive blends to leafhoppers in the tea plant, peach plant, and grapevine volatiles, respectively.

### Development of two leafhopper attractants: formula-P and formula-G

**Figure 3:**
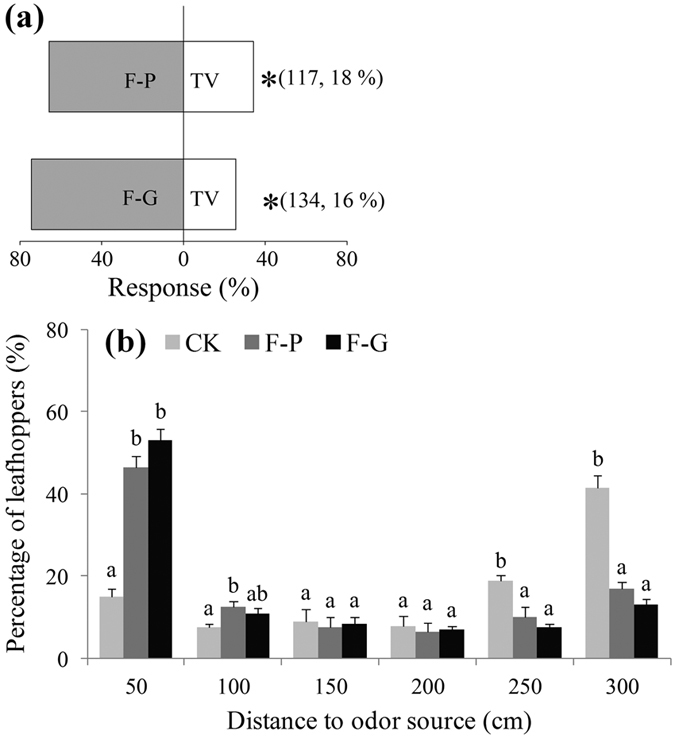
Behavioural responses of *Empoasca onukii* adults in Y-tube (**a**) and wind tunnel (**b**) bioassays to two attractants (F-P and F-G; for composition, see Table 1). In Y-tube bioassay, TV denotes tea plant volatiles, and asterisks denote significant difference in attractiveness (χ^2^ goodness-of-fit test: *P* < 0.0001). Numbers in parentheses indicate numbers of tested leafhoppers followed by frequency (%) of non-responding individuals. In wind tunnel test, percentages of leafhoppers (%, mean + SE) at different distances from odour source were compared among different treatments. In blank control (CK), rubber septa were loaded with liquid paraffin. Each treatment was tested five times. Different letters on bars indicate significant differences (one-way ANOVA, followed by Tukey’s multiple comparison test, *P* < 0.05).

The compositions of formula-P and formula-G are shown in Table 1. In Y-tube tests, the leafhoppers significantly preferred formula-P and formula-G to tea plant odour (*P* < 0.0001) (Fig. 3a). Analyses of the volatiles used in Y-tube tests showed the emission amounts of (Z)-3-hexenyl acetate, DMNT, and (*E*)-ocimene in formula-P and formula-G were similar to those in tea plant volatiles (*P* > 0.05) (Fig. 2d and e). Benzaldehyde in formula-P and ethyl benzoate in formula-G were unique compounds that were not found in tea plant volatiles (Fig. 2d,e).

In the wind tunnel experiments, formula-P and formula-G showed similar attractiveness to leafhoppers (*P* > 0.05) (Fig. 3b). At 50 cm from the odour source, the percentages of leafhoppers attracted to formula-P and formula-G were 46.5% and 53%, respectively, both significantly higher than that in the control (15%, *P* < 0.05) (Fig. 3b). At 250–300 cm from the odour source, the percentages of leafhoppers in the formula-P and formula-G treatments were 17% and 13%, respectively, both significantly lower than that in the control (41.5%, *P* < 0.05) (Fig. 3b).

### Field trapping tests

**Figure 4:**
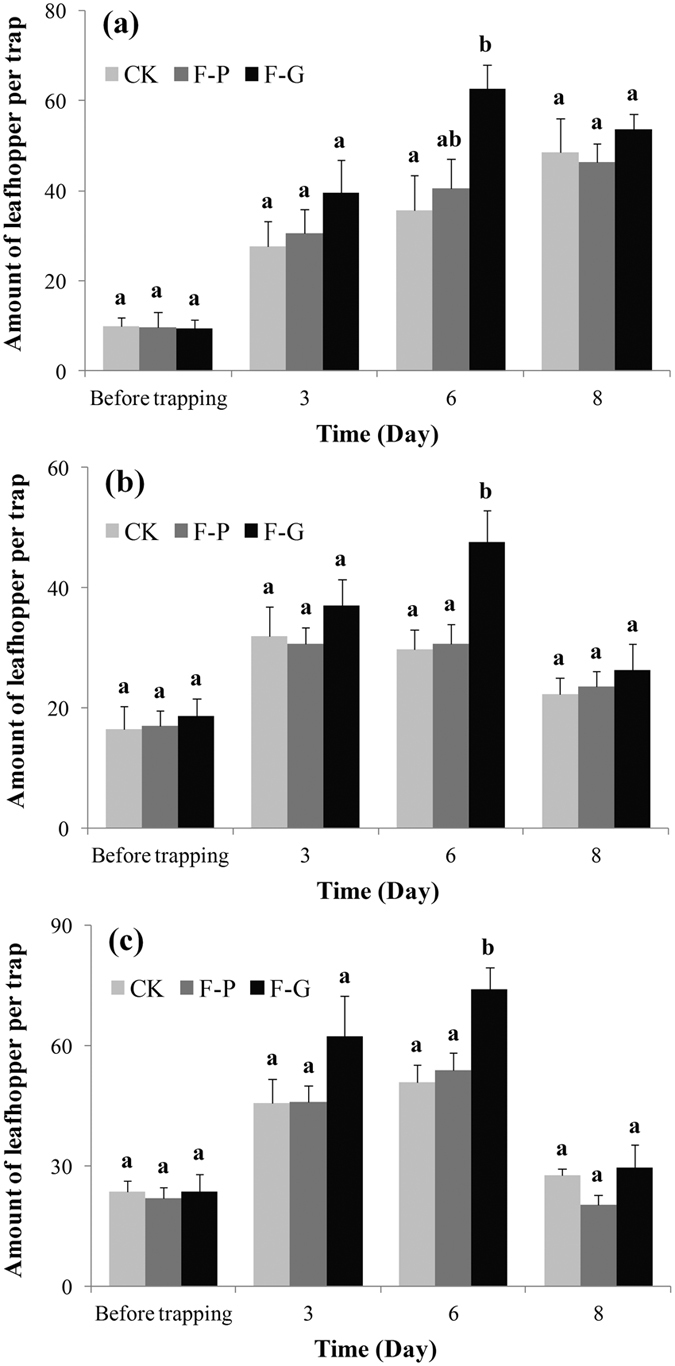
Mean number + SE of *Empoasca onukii* adults captured in each trap at tea plantations in Hangzhou (2014, (**a**); 2015, (**b**)) and Shaoxing (2015, (**c**)). Traps were baited with rubber septa with the two formulae (F-P and F-G; for composition, see Table 1) and a liquid paraffin control (CK). Each treatment was replicated five times. Leafhoppers were trapped on sticky yellow strips placed 1 day before trials. Traps were monitored at 3, 6, and 8 days after laying traps. Different letters on bars indicate significant differences (one-way ANOVA followed by Tukey’s multiple comparison test, *P* < 0.05).

Figure 4 shows the results of the field tests at Hangzhou (in 2014 and 2015) and Shaoxing (in 2015). Before the field trial, the leafhopper populations were at approximately equal densities in the different treatments (*P* > 0.05) (Fig. 4a–c). At 6 days after laying traps, about 1.5–1.7 times more leafhoppers were captured in the formula-G trap than in the control trap (*P* < 0.05) (Fig. 4a–c). At 8 days after laying traps, similar numbers of leafhoppers had been captured by the formula-G and the control traps (*P* > 0.05) (Fig. 4a–c). Similar numbers of leafhoppers were captured by formula-P traps and control traps during the field trial (*P* > 0.05) (Fig. 4a–c).

### Tea plantation background odour

The background odour of tea plantations contained three compounds that were components of formula-P or formula-G: (Z)-3-hexenyl acetate, benzaldehyde, and (*E*)-ocimene (Table 2). Benzaldehyde was detected in all tea plantation air samples at a concentration of approximately 3.5 ng L^−1^, thousands of times higher than the concentrations of the other two detected compounds (Table 2). (E)-Ocimene showed the lowest detection rates (in 47% and 13% of background odour samples at Hangzhou and Shaoxing, respectively) (Table 2).

### Attractiveness of benzaldehyde

**Figure 5:**
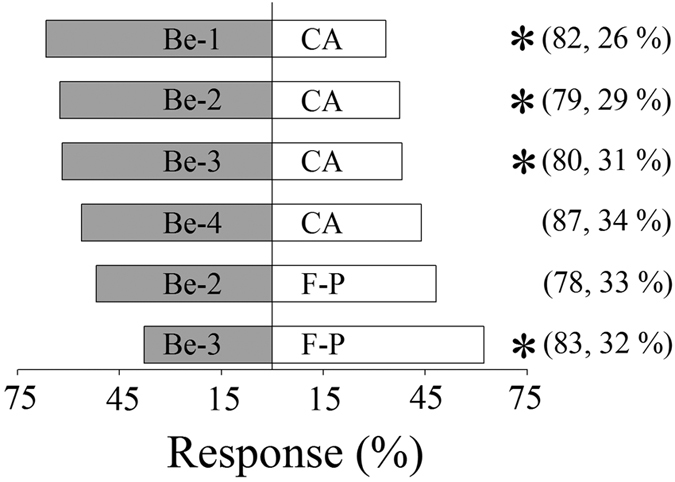
Behavioural responses of *Empoasca onukii* adults in Y-tube bioassay in response to different concentrations of benzaldehyde in air, clean air (CA), or formula-P (F-P). Concentrations of benzaldehyde in air: 790.1 ± 37.1 ng L^−1^ (Be-1), 20.1 ± 4.2 ng L^−1^ (Be-2), 3.4 ± 0.3 ng L^−1^ (Be-3), and 0.4 ± 0.1 ng L^−1^ (Be-4). Benzaldehyde concentration in F-P in air was 350.6 ± 19.1 ng L^−1^. Asterisks denote significant difference in attractiveness (χ^2^ goodness-of-fit test: *P* < 0.05). Numbers in parentheses indicate numbers of tested leafhoppers followed by frequency (%) of non-responding individuals.

The results of the Y-tube tests showed that benzaldehyde in the concentration range of 790.1 to 3.4 ng L^−1^ was significantly more attractive than clean air to leafhoppers (*P* < 0.05) (Fig. 5). Benzaldehyde at various concentrations affected the attractiveness of formula-P to leafhoppers. Compared with benzaldehyde at 3.4 ng L^−1^ in air, the leafhoppers significantly preferred formula-P odour (containing benzaldehyde at 350.6 ng L^−1^) (*P* < 0.05) (Fig. 5). However, when the concentration of benzaldehyde in air was increased to 20.1 ng L^−1^, the leafhoppers could not differentiate between benzaldehyde and formula-P odour (*P* > 0.05) (Fig. 5).

## Discussion

Because the leafhoppers have a small body (body length about 3 mm) and short antennae (<1 mm), it is difficult to screen for the attractive compounds in plant volatiles by GC-electroantennographic detection analysis. Therefore, the attractive blends to leafhoppers in tea plant, peach plant, and grapevine volatiles were identified by Y-tube tests via a process of elimination. The results of the elimination tests could not provide detailed information about the role of every compound in attracting the leafhoppers, but this is a topic worthy of further research. Previous studies have confirmed that the attractive blends in host plant volatiles include at least two types of volatile compounds; green leaf volatile compounds (such as (Z)-3-hexenol and (Z)-3-hexenyl acetate) and terpenes (such as ocimene and DMNT)^3^,^4^. In the present study, (Z)-3-hexenyl acetate was the only green leaf volatile compound present in all three plant volatiles. Therefore, we assumed that it was crucial for attracting leafhoppers, and included it in all the test blends. In laboratory tests, the three reduced blends had similar attractiveness as the three respective plant volatiles. These attractive blends composed of three or four components offered the opportunity to develop an effective leafhopper attractant. In the three blends, some compounds were fundamentally important for attracting leafhoppers. For example, removing ethyl benzoate from blend 8 (to produce blend 9) decreased the attractive efficiency from 88.5% to 68.9%, confirming that ethyl benzoate was fundamentally important for leafhopper attraction in grapevine volatiles. Similarly, blend 5 (blend 4 with DMNT removed) (Table 1) was not attractive to the leafhoppers, indicating that DMNT was a fundamentally important compound in tea plant volatiles. However, our results suggested that DMNT played a minor role as an attractant in peach plant volatiles, because removing DMNT from blend 6 (Table 1) barely affected its attractiveness. This is probably because there was a very low concentration of DMNT in peach plant volatiles.

Two synthetic attractants for leafhoppers were developed by adding benzaldehyde (formula-P) or ethyl benzoate (formula-G) at emission amounts similar to those in peach plant and grapevine volatiles, respectively, to the attractive blend in tea plant volatiles. The Y-tube tests showed that both attractants were significantly more attractive than tea plant volatiles to leafhoppers. Volatile analyses confirmed that benzaldehyde and ethyl benzoate were the main attracting components in formula-P and formula-G, respectively. Then, to simulate the conditions in tea plantations, we used a wind tunnel containing tea plant shoots to test the attractiveness of the synthetic blends. In this assay, the tea plant shoots provided the visual and olfactory background of a tea plantation for the tested leafhoppers. In the wind tunnel bioassay, the two attractants were significantly and similarly attractive to leafhoppers against the background odour of tea plant volatiles.

However, the field trapping trials produced different results. In the tea plantations, the leafhoppers were only significantly attracted to formula-G, which contained ethyl benzoate. This raised the question of why only one of the two attractants that were attractive in the laboratory was attractive to leafhoppers in the tea plantations. We speculated that the odour environment of the tea plantations might have affected the efficiency of the two attractants differently, as a result of their composition. During the field tests, three components ((*Z*)-3-hexenyl acetate, (*E*)-ocimene, and benzaldehyde) of the two attractants were detected in the ambient air of the tea plantation. The concentration of benzaldehyde in tea plantation air was thousands of times higher than the concentrations of the other two detected compounds. The high concentration of benzaldehyde in the tea plantation background odour would strongly disrupt the attractive efficiency of formula-P, which had benzaldehyde as its main component. The Y-tube bioassay showed that benzaldehyde at the concentration detected in tea plantation background odour was attractive to leafhoppers. This bioassay also showed that the leafhoppers did not distinguish between benzaldehyde and formula-P when the concentration of benzaldehyde in formula-P was less than about 20 times higher than that of benzaldehyde in air. In field conditions, the concentration of an odour decreases with increasing distance from its source^1^. When the concentration of benzaldehyde from formula-P decreased to a similar level as that in the tea plantation background odour, the leafhoppers could not differentiate between benzaldehyde from the two sources. Our unpublished data indicated that the concentration of benzaldehyde in the tea plantation ambient air at 1 m from the formula-P attractant was similar to that in the control plot. Therefore, the high concentration of benzaldehyde in the ambient air of the tea plantation could lead to the very small control range of point-released formula-P. More experiments should be conducted to further investigate the effects of a high concentration of benzaldehyde in the background odour on the attractiveness of formula-P. Besides benzaldehyde, (Z)-3-hexenyl acetate, (*E*)-ocimene, and DMNT were also detected in the background odour of the tea plantations. However, they were detected at very low concentrations, and were minor components of the two attractants. Therefore, we assumed that these compounds in the ambient air of the tea plantations had little effect on the trapping efficiency of the two attractants.

The field background odour includes the volatiles from the main crop and other plants, as well as volatiles from motor vehicles and nearby industries. Our previous studies showed that tea plants infested by tea geometrid (*Ectropis obliqua*), tea weevil (*Myllocerinus aurolineatus*), and leafhoppers emitted large amounts of (*Z*)-3-hexenyl acetate, (*E*)-ocimene, and DMNT^41^,^42^. However, benzaldehyde was only emitted from the tea plants severely infested by tea geometrids and tea weevils, and its emission amount was tens of times lower than that of (*E*)-ocimene^41^,^42^. Thus, the high concentration of benzaldehyde detected in the tea plantation background odour in this study would not have originated from the tea plants. Benzaldehyde is a common toxic volatile compound in the atmosphere, and it can originate from motor vehicle and industrial emissions^43^,^44^. It is likely that these were the main sources of benzaldehyde in the tea plantation background odour. Because field background odours are more complex than crop volatiles, the components of an attractant that differ from those of main crop volatiles may not differ from those of the field background odour.

An essential factor for insect olfactory location is that the attractant must stand out from the background volatile noise. There are two ways to avoid disturbance by background odour^31^: (1) increase the amount of the attractant, or (2) increase the specificity of the attractant. If the composition of the attractant overlaps with that of the background odour, then increasing its amount will only slightly increase its detectability. However, if the components of the attractant differ from those of the background odour, then even a small amount of the attractant will be highly detectable. Therefore, the specificity of attractant’s composition relative to that of the background odour is crucial for its attractiveness to the target pest in field conditions.

The long-term goal of our research is to develop the synthetic attractants based on plant volatiles to monitor and control tea leafhopper in tea plantations. In this study, we developed two formulae that differed from tea plant volatiles, and were more attractive than tea plant volatiles to leafhoppers in laboratory tests. However, one of them was not specific relative to the background odour of the tea plantation, and only the attractant with a specific component that stood out from the tea plantation background odour was attractive to leafhoppers in the field. These results indicated that a synthetic attractant should contain components that not only differ from those of the main crop, but also from those of the field background odour. Further research to develop a safe and effective method to monitor and control leafhoppers should focus on how to enhance the efficiency of formula-G in field conditions, and how to efficiently kill the attracted leafhoppers.

## Methods

### Plants, insects, and chemicals

The tea plants (*Camellia sinensis* cv. ‘clone Longjing 43’), peach plants (*Prunus persica* cv. ‘Hongtiantao’ (Rosaceae)), grapevine plants (*Vitis vinifera* cv. ‘Kyoho’ (Vitaceae)), and tea leafhoppers (*Empoasca onukii*) used in this study were prepared as described previously^40^. Test compounds (Table 1) were high-purity grade, and were obtained from Sigma-Aldrich (China) except for DMNT, which was obtained by custom synthesis. All compounds were dissolved in liquid paraffin to different concentrations for Y-tube tests and wind tunnel bioassays, or in methanol for volatile analyses. Pure synthetic compounds were used in the field trapping tests.

### Y-tube bioassay

#### Odour sources

Plant odour sources were prepared as described previously^40^. Synthetic compounds in liquid paraffin were mixed as shown in Table 1, and 20 μL solution of synthetic compounds or 20 μL liquid paraffin (clean air) was loaded onto each rubber septum (8 mm O.D. Sigma-Aldrich, China). According to the three plant volatiles^40^, the total loaded amount of the synthetic compounds was 0.1–0.6 ng per rubber septum.

#### Y-tube setup and bioassays

The Y-tube hardware and the bioassay procedure were as described previously^40^. The proportions of tested male and female leafhoppers were random. The Y-tube and odour sources were replaced after testing 10 individuals. Bioassays were conducted between 15:00 and 19:00 h. On a given day, approximately 30 leafhopper individuals were tested, and each insect was used only once.

#### Treatments

To identify the attractive blends to leafhoppers among the volatiles of peach plants, grapevine, and tea plants, 11 blends (Table 1) of compounds were tested. The tea plant, peach plant, and grapevine volatiles shared five common compounds ((*Z*)-3-hexenyl acetate, (*E*)-ocimene, linalool, DMNT, and (*E*,*E*)-α-farnesene), and the tea plant volatile blend was the simplest of the three plant volatile blends^40^. The attractive blend in tea plant volatiles was first identified, and the starting point was a blend of the five common compounds. Each blend and clean air or plant volatiles were provided as choices for the leafhoppers. Next, according to the composition of the tea plant volatiles, the emission amounts of (Z)-3-hexenyl acetate, DMNT, and (*E*)-ocimene in blend 6 and 10 were adjusted to create two attractants (formula-P and formula-G). In formula-P, the relative ratio of (Z)-3-hexenyl acetate, (*E*)-ocimene, DMNT, and benzaldehyde was 1.5:1.9:1.0:8.1, and the total loaded amount of synthetic compounds was 0.2 mg. In formula-G, the relative ratio of (Z)-3-hexenyl acetate, (*E*)-ocimene, DMNT, and ethyl benzoate was 10.0:12.5:6.7:1.0, and the total loaded amount of synthetic compounds was 0.6 mg. To validate their attractiveness to the leafhoppers, each of the attractants was compared with tea plant volatiles. Finally, to explore the attractiveness of benzaldehyde, four different concentrations of benzaldehyde in air (790.1, 20.1, 3.4 and 0.4 ng L^−1^) were compared with clean air or formula-P. In this experiment, the odour sources were replaced after testing five individuals. In total, there were 22 treatments in the Y-tube bioassays. Each treatment was repeatedly tested for 3–4 days on approximately 100 adult leafhoppers. All Y-tube tests were completed within 80 days.

### Wind tunnel test

#### Hardware

**Figure 6:**
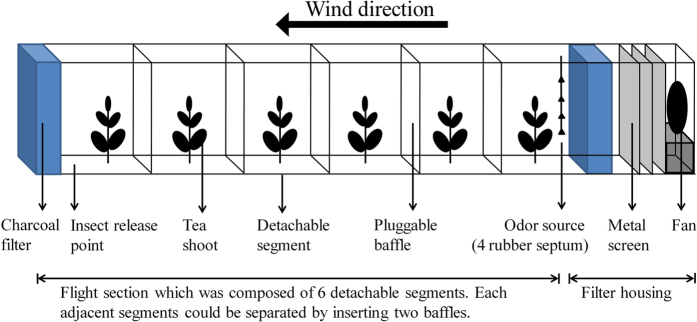
Schematic illustration of wind tunnel used to test leafhopper attraction to odours.

Leafhopper attraction was tested in a wind tunnel with a polycarbonate flight section (25 × 20 × 300 cm) consisting of six detachable segments, each 50 cm long (Fig. 6). Adjacent segments could be separated by inserting two baffles. Four tea shoots (the same as those used in the Y-tube test) in floral foam soaked with water were placed in the centre of each segment. Air was blown into the tunnel by a mini fan (15 cm high, 12 cm wide, LSF95, Lisuo, China) through three metal screens (100 mesh, 5 cm distance between screens) and a box (25 × 20 × 5 cm) filled with active charcoal. The air leaving the tunnel passed through a 100-mesh metal screen and a box filled with active charcoal before being released back into the room. The wind speed was calibrated to 20 cm s^−1^ by a hot-film anemometer (AR866, Dongguan Science & Technology Co. Ltd., Dongguan, China).

#### Odour sources

In the wind tunnel experiments, 6 mg synthetic formula-P or 2 mg synthetic formula-G in liquid paraffin was loaded onto four rubber septa, which were threaded onto a string approximately 12 cm long with paper clips. The string was hung at the upwind end of the flight section of the tunnel. There was a 4-cm distance between the uppermost rubber septum and the top of the wind tunnel. In the blank control, the rubber septa were loaded with liquid paraffin.

#### Test protocol

The wind tunnel was lit diffusely from above at about 200 lux. The room was kept at 20 ± 2 °C, 70–80% R.H. Before the test, the flight sections were cleaned with ethyl alcohol and maintained in a ventilated environment for 8 h. The leafhoppers were the same as those used in the Y-tube test. Forty leafhoppers were placed in a 5-ml centrifuge tube and transferred to the downwind end of the wind tunnel at 16:00. All lights in the wind tunnel room were turned off 2 h after releasing the leafhoppers. After 6 h (at 00:00 on day 2), baffles were inserted between the segments of the wind tunnel, and the number of leafhoppers in each segment was counted. Each treatment was tested five times using 200 leafhoppers in total. Different treatments were tested on 3 consecutive days, and all of the wind tunnel tests were completed within 20 days.

### Field trapping tests

To test the attractiveness of the two formulae to leafhoppers, field tests were conducted in October at two tea plantations: a 300-hectare tea plantation at the Tea Experimental Plantation of the Tea Research Institute (Chinese Academy of Agricultural Sciences, Hangzhou, China) in 2014 and 2015; and a 200-hectare tea plantation at the Ming Shan Tea Factory (Shaoxing, China) in 2015. The cultivar in both tea plantations was clone Longjing 43. The field trials included three treatments (formula-P, formula-G, and a blank control), and each treatment had five replicates. The 15 experimental plots were in a complete randomized design in each field trial. In each replication, 360 mg synthetic formula-P was loaded onto nine rubber septa, 120 mg synthetic formula-G was loaded onto three rubber septa, and nine blank rubber septa were used in the control. The rubber septa were mounted in a disposable paper cup, which was suspended upside-down at 0.10 m below the tea bush canopy. To monitor leafhoppers, a sticky yellow trap (25 × 20 cm, Enjoy Technology, China) was placed approximately 0.35 m above each disposable paper cup^45^. One trap was used in each replication, and the distance between traps was at least 30 m to minimize interference between lures. To confirm that the leafhopper populations were at approximately equal densities in the different treatments, sticky yellow traps were placed at the trap sites 1 day before the trials. The traps were monitored at 3, 6 and 8 days after the start of the experiment. The sticky yellow traps were replaced on each survey day.

### Volatile collection and analysis

#### Release of plant and synthetic volatiles in Y-tube tests

Volatiles were collected and analysed as described previously^40^. The plant shoots and rubber septa loaded with synthetic compounds were maintained in the holding chambers as in Y-tube tests. The collection lasted 0.5 h. Volatile collection for each odour source was replicated four times. After sampling, the traps were extracted with 500 mL methylene chloride, and 50 ng ethyl decanoate (internal standard) was added to the extract. The volatiles were analysed by gas chromatography-mass spectrometry (GC-MS) using a QP2010 GS-MS instrument (Shimadzu, Japan).

#### Tea plantations background odour

During the field trapping tests, samples of tea plantation background odour were collected from all experimental plots (one sample per plot) 1 day before trapping, and from all control plots on days 3, 6, and 8 after the field trapping tests started. In total, 120 samples were collected at Hangzhou and Shaoxing in 2014 and 2015. The plant volatile compounds in ambient air at tea plantations were collected and analysed as described by Cai *et al*.^46^. Air near the tea bush canopy was collected at a flow rate of 100 mL min^−1^ for 240 min (24-L samples) using a microprocessor-controlled air sampling pump (Mini-pump Σ30; Shibata, Japan) at 13:00 h. After collection, all samples were taken to the laboratory and analysed immediately. All field samples were spiked with 5 ng ethyl decanoate (internal standard), and were analysed by a coupled thermal desorption (TD; TD100, Marks, UK) GC-MS (GCMS-QP2010, Shimadzu, Japan).

### Statistical analysis

All statistical tests were carried out using SAS v8.2 (SAS Institute, Cary, NC, USA). For the olfactometer test, the null hypothesis that *E. onukii* showed no preference for either olfactometer arm (a response equal to 50:50) was analysed with a χ^2^ goodness-of-fit test after correcting for continuity with Yates’ correction factor^47^. Differences in the amounts of volatile compounds emitted from odour sources in the Y-tube test were determined using two-sample t-test for means. Mean percentages of leafhoppers in different segments of the wind tunnel were analysed by one-way ANOVA. In field trapping tests, mean numbers of leafhoppers in different treatments before trapping and at 3, 6, and 8 days after laying traps were analysed by one-way ANOVA. Mean values were separated by Tukey’s multiple range tests (*P* < 0.05).

## Additional Information

**How to cite this article**: Cai, X. *et al*. Field background odour should be taken into account when formulating a pest attractant based on plant volatiles. *Sci. Rep.* **7**, 41818; doi: 10.1038/srep41818 (2017).

**Publisher's note:** Springer Nature remains neutral with regard to jurisdictional claims in published maps and institutional affiliations.

## Figures and Tables

**Table 1:** Composition of 11 blends (B-1 to B-11) to detect attractive blends to adult *Empoasca onukii* in tea plant, peach plant, and grapevine volatiles, and composition of two attractants (F-P and F-G) for trapping *E. onukii*.

Volatile compounds	Tea plant					Peach plant		Grapevine				Lure	
	B-1	B-2	B-3	B-4	B-5	B-6	B-7	B-8	B-9	B-10	B-11	F-P	F-G
HA	•	•	•	•	•	•	•	•	•	•	•	•	•
HB								•	•		•		
Oc	•	•		•	•	•	•	•	•	•	•	•	•
Li	•	•	•										
DM	•	•	•	•		•		•	•	•		•	•
Fa	•												
Be						•	•					•	
EB								•		•	•		•

Tea plant, peach plant, and grapevine volatiles have been described in detail by Cai *et al*.^40^. HA, (Z)-3-hexenyl acetate; HB, (Z)-3-hexenyl butyrate; Oc, (*E*)-ocimene; Li, Linalool; DM, (E)-4,8-dimethyl-1,3,7-nonatriene; Fa, (*E, E*)-α-farnesene; Be, benzaldehyde; EB, ethyl benzoate.

**Table 2:** Concentrations and detection rates of three components of two attractants in background odour of tea plantation.

Compounds	Detection rate (%)		Concentration (mean ± SE, ng L^−1^)	
	Hangzhou	Shaoxing	Hangzhou	Shaoxing
(*Z*)-3-Hexenyl acetate	100	80	0.004 ± 0.001	0.003 ± 0.001
(*E*)-Ocimene	47	13	0.001	0.001
Benzaldehyde	100	100	3.334 ± 0.135	3.674 ± 0.201

Sampling was performed in all experiment plots 1 day before the field trapping test, and in control plots on days 3, 6, and 8 days after start of field trapping test. Air near tea bush canopy was collected at 100 mL min^−1^ for 240 min (24-L samples). Detection rate is detection frequency of a volatile compound in all samples at Hangzhou or Shaoxing.
